# Evaluation Strategies for Large Language Model-Based Models in Exercise and Health Coaching: Scoping Review

**DOI:** 10.2196/79217

**Published:** 2025-10-14

**Authors:** Xiangxun Lai, Yue Lai, JiaCheng Chen, Shengqi Huang, Qi Gao, Caihua Huang

**Affiliations:** 1 Research and Communication Center for Exercise and Health, Xiamen University of Technology, 600 Ligong Road, Jimei District, Xiamen, Fujian Province, 361024, China, 86 15606951380; 2 School of Sport Medicine and Rehabilitation, Beijing Sport University, Beijing, China; 3 Department of Mathematics and Digital Science, Chengyi College, Jimei University, Xiamen, China

**Keywords:** large language models (LLMs), evaluation metrics, coaching, exercise, movement, scoping review

## Abstract

**Background:**

Large language model (LLM)-based artificial intelligence (AI) coaches show promise for personalized exercise and health interventions. However, the unique demands of ensuring safety and real-time, multimodal personalized feedback have created a fragmented evaluation landscape lacking standardized frameworks.

**Objective:**

This scoping review systematically maps current evaluation strategies for LLM-based AI coaches in exercise and health, identifies strengths and limitations, and proposes directions for robust, standardized validation.

**Methods:**

Following PRISMA-ScR (Preferred Reporting Items for Systematic reviews and Meta-Analyses extension for Scoping Reviews) guidelines, we conducted a systematic search across 6 major databases (eg, PubMed, Web of Science) for original research on LLM-based exercise and health coaching. Studies were included if they explicitly reported on evaluation methods. We extracted and synthesized data on model types, application domains, and evaluation strategies and developed a 5-point Evaluation Rigor Score (ERS) to quantitatively assess the methodological depth of the evaluation designs.

**Results:**

We included 20 studies, most using proprietary models like ChatGPT (75%). Evaluation strategies were highly heterogeneous, mixing human ratings (80%) and automated metrics (40%). Crucially, the evidence was limited by low methodological rigor: the median ERS was 2.5 out of 5, with 55% of studies classified as having low rigor. Key gaps included limited use of real-world data (40%) and inconsistent reliability reporting (45%).

**Conclusions:**

The current evaluation of LLM-based health coaches is fragmented and methodologically weak. Future work must establish multidimensional validation frameworks that integrate technical benchmarks with human-centered methods to ensure safe, effective, and equitable deployment.

## Introduction

### Rationale

The advancement of large language models (LLMs) offers considerable potential for developing personalized health interventions. In the domain of exercise and health coaching, these artificial intelligence (AI) systems are expected to perform complex tasks, including generating adaptive training plans, providing real-time movement feedback, and offering motivational support [1,2]. However, translating this potential into safe and effective applications presents unique and substantial challenges. Effective exercise coaching requires the dynamic integration of multimodal data streams—such as textual user reports, visual posture analysis, and physiological sensor data—while ensuring clinical safety and a high degree of personalization [3,4]. This complexity demands rigorous and specialized evaluation methods.

Establishing trust and ensuring efficacy in this high-stakes domain hinges critically upon robust performance evaluation. While standardized benchmarks are instrumental for assessing general LLMs (eg, MMLU, HumanEval [OpenAI]) and in knowledge-intensive fields like medicine [5], these frameworks are ill-equipped to address the unique demands of exercise coaching. This limitation persists even with the emergence of benchmarks for the broader sports domain. For instance, knowledge-based benchmarks like SportQA can assess an AI’s understanding of rules, and multimodal benchmarks like SCBench or SPORTU can evaluate its ability to describe actions in a sports video [6-8]. However, these descriptive and knowledge-retrieval tasks are fundamentally different from the prescriptive, safety-critical, and personalized nature of coaching. None of these frameworks can evaluate an AI’s ability to provide safe, real-time corrective feedback on a user’s squat form or to dynamically adapt a workout plan based on reported fatigue, highlighting a critical gap in evaluation methodology [9,10].

Consequently, the current evaluation landscape for AI exercise coaches is notably heterogeneous and lacks standardization [11-13]. A review of existing literature reveals a fragmented approach: some studies focus on quantitative metrics like movement classification accuracy, others rely on user surveys to measure subjective engagement and usability, while a third group relies on expert panels to assess the safety and appropriateness of generated plans. This fragmentation creates a critical bottleneck, hindering the systematic comparison of different AI coaching systems and impeding evidence-based, iterative improvement. Without a clear understanding of how these systems are currently being evaluated—including the strengths and weaknesses of existing methods—the field cannot move toward developing the robust, multidimensional validation frameworks it urgently needs.

Given the nascent and fragmented nature of this field, a scoping review was chosen as the most appropriate methodology over a systematic review. The primary objective is not to answer a narrow question about the efficacy of a specific LLM coach, but rather to map the breadth and diversity of existing evaluation strategies, identify key concepts and evidence gaps, and synthesize findings from a heterogeneous body of literature. This approach is ideally suited for clarifying the scope of this emerging research area and informing the development of future standardized evaluation frameworks.

### Objective

Amidst the growing yet fragmented use of LLM-based AI coaches across exercise, physical activity, fitness, sports, and rehabilitation, this scoping review aims to:

1. Identify evaluation methods used to assess these AI coaches in health and exercise settings;
2. Summarize the strengths, limitations, and validation approaches (eg, user feedback, expert ratings, real-world testing);
3. Develop a conceptual framework to guide future evaluations;
4. Highlight key gaps and directions for future research.

This synthesis aims to support more rigorous and standardized validation of next-generation AI coaching tools.

## Methods

### Design

We followed the scoping review framework outlined by Arksey and O’Malley [14], which includes:(1) identifying the research question, (2) identifying relevant studies, (3) selecting studies, (4) charting the data, and (5) collating, summarizing, and reporting the results. We also adhered to the PRISMA-ScR (Preferred Reporting Items for Systematic reviews and Meta-Analyses extension for Scoping Reviews) checklist to ensure transparency and methodological rigor throughout the review process [15].

### Search Strategy Development and Study Selection

The search strategy was developed collaboratively by 2 reviewers (XL and CH), drawing on previous literature and expert input on the evolving landscape of LLM applications in health and exercise science. To ensure consistency in the application of the eligibility criteria, a pilot test was conducted on 10 articles at the full-text screening stage. This pilot phase was crucial for operationalizing our inclusion criteria. For example, we identified initial ambiguity in applying the criterion “Performance evaluation.” During the pilot, it became apparent that a clearer definition was needed to distinguish between studies that merely described an LLM’s output versus those that formally evaluated it. To resolve this, the pilot test led us to refine this criterion to be more specific: "The article explicitly reported at least one strategy to evaluate model performance, such as accuracy, expert scoring, user feedback, usability studies, or benchmarking.” This clarification was instrumental in standardizing the screening process and ensuring that both reviewers applied the criteria consistently.

Systematic searches were conducted in 6 major databases: PubMed, Web of Science, Google Scholar, arXiv, medRxiv, and bioRxiv. The search strategy combined three core conceptual domains using Boolean logic: (1) large language models, (2) exercise and health coaching (including terms for physical activity, rehabilitation, fitness, and sports), and (3) evaluation (including terms for performance, metrics, and benchmarks). The complete, unabridged search strategies for each database, including the exact search strings, search dates, and all applied filters, are provided in Multimedia Appendix 1. To supplement the database search, a “snowballing” technique was also used [16], where the reference lists of included articles were manually screened for additional relevant studies.

### Eligibility Criteria

The specific inclusion and exclusion criteria used for the study selection process are presented in Textbox 1.

This textbox details the specific criteria applied during the study selection process. The criteria were designed to identify original research articles focused on the evaluation of LLM-based coaching systems within the exercise and health domain, ensuring a focused and reproducible literature search. Notably, for the “Model type” criterion, we adopted a functional rather than a purely architectural definition. We included studies that either explicitly named a specific LLM (eg, GPT-4 [Open AI]) or described core system capabilities, such as the generation of novel, unscripted, and context-aware conversational feedback, which are hallmarks of modern generative AI and distinguish them from traditional rule-based systems. This approach allows for the inclusion of early or hybrid systems that leverage LLM capabilities, even if they incorporate rule-based fallbacks or do not detail the specific underlying architecture, reflecting the real-world heterogeneity of this rapidly emerging field.

Textbox 1. **Inclusion and exclusion criteria for study selection.**

**Inclusion criteria**:

1. Language: published in English.
2. Model type: the study describes a system intended for exercise and health coaching that explicitly identifies its core technology as a large language model (LLM; eg, GPT, Llama) or describes functionalities strongly indicative of generative AI capabilities (eg, generating novel and unscripted conversational responses).
3. Domain relevance: focused on exercise, physical activity, rehabilitation, or sport.
4. Performance evaluation: the article explicitly reported at least one strategy to evaluate model performance, such as accuracy, expert scoring, user feedback, usability studies, or benchmarking.
5. Original research: the study contained original experimental design, results, or evaluation methods.

**Exclusion criteria**:

1. Studies not peer-reviewed or not published in English.
2. Nonoriginal research content, such as reviews, abstracts, letters, viewpoints, editorials, dissertations, and tutorials (unless they provided original research data).
3. Articles that did not clearly describe any method to assess model evaluation.
4. Studies not related to exercise, fitness, sports, or physical activity movement coaching (eg, those focused on education or psychology).
5. Studies describing systems based solely on traditional AI, such as rule-based engines, decision trees, or classic machine learning classifiers, without any mention of generative or LLM components.

### Data Charting Process

All search results were imported into EndNote 2025, and duplicates were removed. Study selection was conducted in 2 stages—title and abstract screening and full-text screening—followed by data charting. Both screening and charting processes were independently performed by 2 reviewers (XL and CH). To ensure reliability, interrater agreement was calculated on a random sample of 50 titles and abstracts, yielding a Cohen Kappa score of 0.88, indicating substantial agreement. Any disagreements at any stage were first discussed between the 2 reviewers; if unresolved, a final decision was made by a third reviewer (QG). This rigorous and consistent process ensured the validity and reliability of our findings and reinforced the overall quality of the review.

### Data Items

The data extraction categories for included studies are summarized in Textbox 2.

Textbox 2. **Data extraction categories for included studies.**

For each included study, the following information was extracted:

- Author name and publication date: study reference details.
- Model name: name or identifier of the proposed system.
- Application scenario: the context in which the model was applied within the exercise or health domain (eg, fitness coaching, rehabilitation, motion correction).
- Basic model: the underlying large language model (LLM) or architecture used (eg, GPT-4 [Open AI]).
- Input type: format of inputs received by the model (eg, prompts, self-tracking data, video frames).
- Output type: type of outputs generated by the model (eg, text advice, motion scores, feedback).
- Datasets: datasets used for training or testing the model.
- Evaluation metrics: performance metrics used to assess the model, encompassing:
  - Automated performance metrics: Objective, computationally derived metrics used to assess model performance (eg, classification accuracy, *F*_1_-score, MAE, benchmarks, text quality measures such as readability scores or Bilingual Evaluation Understudy [BLEU]).
  - Human-rating metrics: subjective metrics based on feedback and scores from human evaluators (eg, expert ratings using Likert scales or Kappa, user feedback).
  - Study-design metadata: information describing the high-level structure, context, and comparative nature of the evaluation (eg, comparisons vs experts or baselines, data and context such as real-world datasets or input modality, and evaluation paradigm such as user study or longitudinal assessment).
- Evaluation outcome: key results related to model performance.

### Quality Assessment of Evaluation Methodologies

While a formal risk-of-bias assessment is not typically required for scoping reviews, our objective to synthesize the rigor of current evaluation methods necessitated a structured approach to quality appraisal. Recognizing that established appraisal tools for traditional study designs are ill-suited for this novel technological domain, we developed and applied a custom scoring system to provide a transparent and consistent measure of the methodological depth of the included studies.

To this end, we created a 5-point Evaluation Rigor Score (ERS). The 5 criteria of the ERS were derived from foundational principles of high-quality research identified in related fields such as human-computer interaction and clinical validation studies. Each criterion represents a key aspect of robust evaluation design, such as the use of real-world data and the inclusion of comparative benchmarks. This structured approach allows for a more nuanced interpretation of the evaluation landscape, distinguishing between preliminary explorations and more robust validation studies. The 5 criteria are as follows:

1. Validation context: studies received 1 point for conducting evaluations in a real-world or simulated user setting, versus 0 points for purely hypothetical scenarios.
2. Data source: studies using real user-generated data (eg, sensor data, interviews) for evaluation were awarded 1 point, versus 0 points for using synthetic or author-generated data.
3. Instrument validity: studies using validated scales (eg, MITI [Motivational Interviewing Treatment Integrity] and SASSI [Subjective Assessment of System Suitability for Implementation]) for human-rating metrics received 1 point, versus 0 points for using unvalidated or custom-developed questionnaires.
4. Interrater reliability: for studies involving subjective coding or expert scores, 1 point was awarded if an interrater reliability metric (eg, Cohen Kappa, Intraclass Correlation Coefficient [ICC]) was reported, versus 0 points if not.
5. Comparative standard: studies including a direct comparison against a baseline (eg, another model, human expert) received 1 point, versus 0 points for standalone model evaluations.

Two reviewers (XL and CH) independently applied the ERS to all included studies. Any discrepancies were resolved through discussion with a third reviewer (QG). The final score for each study, ranging from 0 to 5, is presented in the results section and serves as a quantitative indicator of its methodological rigor.

### Ethical Considerations

This scoping review was approved by the Institutional Review Board of Beijing Sport University (approval number: 2025336H). As the study was based exclusively on the analysis of publicly available data from previously published literature, the requirement for individual participant consent was waived.

## Results

### Overview of Included Studies

Our scoping review identified 20 studies [3,4,11,12,17-32] that met the inclusion criteria, all published within a narrow time frame from March 2023 to July 2025, highlighting the rapid emergence of this research area. The following sections provide a detailed synthesis of these studies, covering the selection process, methodological rigor, key characteristics, and an aggregated overview of the models and evaluation strategies used.

### Identification of Studies

**Figure 1.**
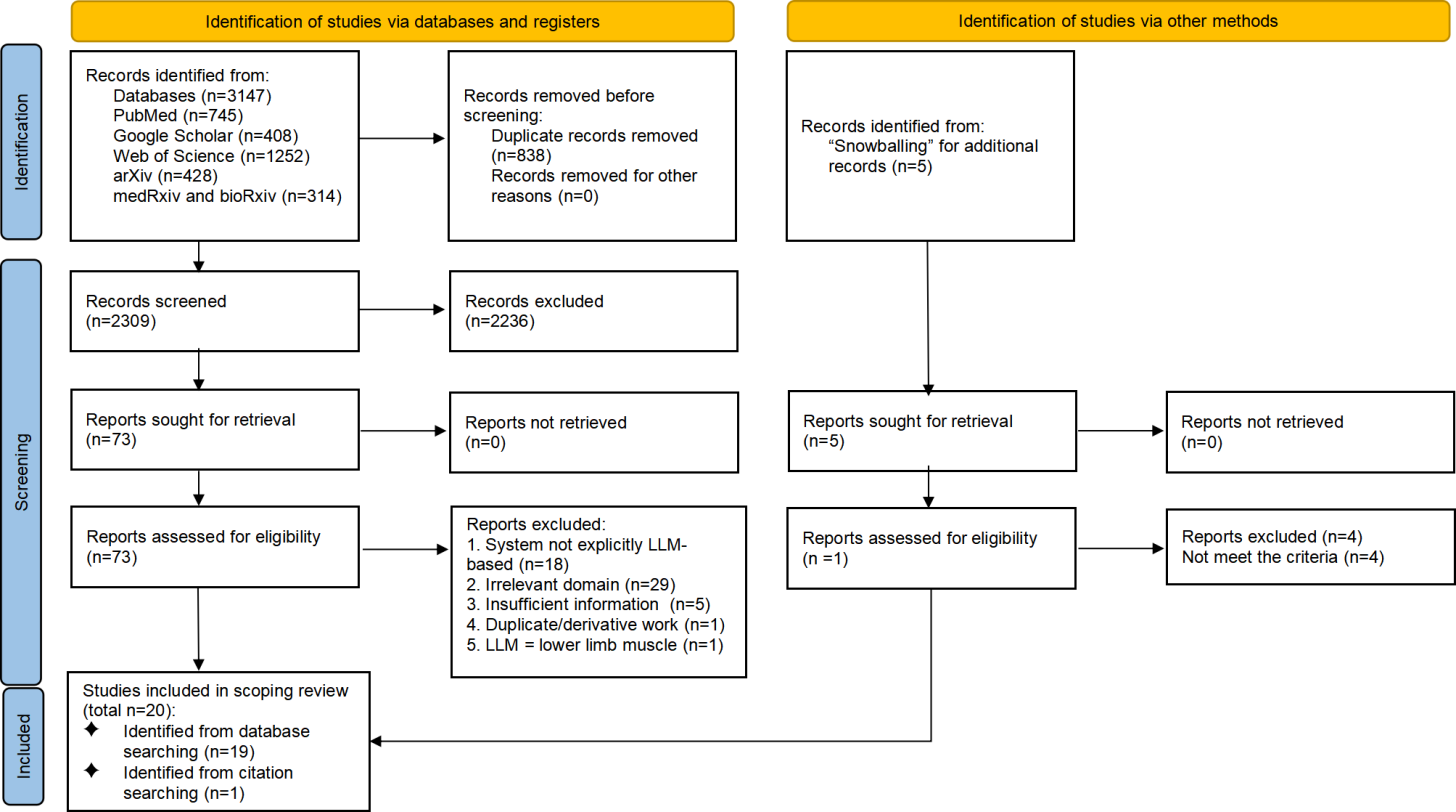
Preferred Reporting Items for Systematic reviews and Meta-Analyses extension for Scoping Reviews (PRISMA-ScR) flow diagram.

The study identification and selection process is detailed in the PRISMA-ScR flow diagram (Figure 1).

The diagram details the study identification and selection process. Our systematic search of 6 databases yielded 3147 records, and 5 additional studies were identified through citation searching (snowballing) based on the reference lists of included articles. After removing duplicates, 2309 records remained for title and abstract screening. Of these, 2236 were excluded, and 73 full-text articles were assessed for eligibility. At the full-text screening stage, 54 articles were excluded: 18 did not involve an LLM-based AI system, 29 were unrelated to exercise, sport, fitness, physical activity, or health coaching, 5 lacked sufficient methodological or evaluative detail, 1 was a duplicate or earlier version of an included study, and 1 misinterpreted “LLM” as “lower limb muscle.” This rigorous screening process led to the final inclusion of 20 studies in this review.

### Methodological Rigor of Evaluation Strategies

To provide a deeper synthesis of the evaluation landscape, we assessed the methodological rigor of these 20 studies using our ERS. The ERS scores revealed considerable heterogeneity in methodological depth, ranging from 1 to 5 with a median score of 2.5, indicating a general trend toward less rigorous evaluation designs (see Table 1). A small minority of studies (2/20, 10%) [17,24] achieved the maximum score of 5, signifying a comprehensive and robust evaluation methodology. In stark contrast, the majority of the literature (11/20, 55%) [3,4,11,12,18,21,22,25,26,28,31] received a low-rigor score of 1 or 2, while the remaining 7 studies [19,20,23,27,29,30,32] (35%) fell into a moderate-rigor category with scores of 3 or 4. Common reasons for lower scores included a lack of real-world validation context and the omission of reliability metrics. For instance, fewer than half of the studies (9/20, 45%) [4,11,17,23,24,27-30] reported interrater reliability for their subjective assessments. Furthermore, only 40% (8/20) [4,17,19,20,24,29,30,32] of the studies situated their evaluation in a real-world or simulated user context, and an equal proportion (8/20, 40%) [3,12,18,21,23,25,26,31] based their evaluations on real user-generated data. This quantitative analysis reinforces our primary finding that the evaluation landscape for LLM-based coaches is not only fragmented in its choice of methods but also highly variable in its methodological rigor.

**Table 1.** Assessment of methodological rigor of included studies using the Evaluation Rigor Score.

Studies	Validation context	Data source	Instrument validity	Interrater reliability	Comparative standard	Total ERS^a^
Jörke et al [17]	1	1	1	1	1	5
Li et al [3]	0	0	1	0	1	2
Xing et al [4]	0	1	0	1	0	2
Ma et al [18]	0	1	0	0	1	2
Yao et al [19]	1	0	1	0	1	3
Mantena et al [20]	1	1	0	0	1	3
Huang et al [21]	1	1	0	0	1	2
Saraç et al [22]	0	0	0	0	1	1
Havers et al [11]	0	0	0	1	1	2
Ong et al [23]	0	1	0	1	1	3
Strömel et al [24]	1	1	1	1	1	5
Kim et al [25]	0	1	1	0	1	2
Willms et al [26]	0	0	1	0	0	1
Zaleski et al [27]	0	0	1	1	1	3
Dergaa et al [28]	0	0	1	1	0	2
Haag et al [29]	1	0	1	1	1	4
Washif et al [12]	0	0	1	0	1	2
Shin et al [30]	1	0	1	0	1	3
Vardhan et al [31]	1	0	0	0	1	2
Sivarajkumar et al [32]	0	1	1	0	1	3

a ERS: Evaluation Rigor Score.

Each included study was scored from 0 to 5 based on 5 criteria for methodological rigor: validation context (1=real-world/simulated user setting), data source (1=real user data), instrument validity (1=used validated scales), interrater reliability (1=reported IRR metrics), and comparative standard (1=included a baseline/expert comparison). The ERS provides a quantitative summary of the methodological depth for each study.

### Characteristics of Included Studies

The key characteristics of the 20 included studies are detailed chronologically in Table 2. The research demonstrates a wide variety of application scenarios, from generating personalized exercise plans [21,30] and prescribing strength programs [11,12] to providing real-time movement analysis [3,4] and supporting cardiac rehabilitation [29]. The studies also used a diverse range of input types, from simple text-based prompts to complex multimodal data streams, including 3D motion capture, video, and wearable sensor data. The outputs were similarly varied, encompassing not only textual advice and training plans but also quantitative scores, explanations, and visualizations.

Table 2 presents a comprehensive summary of 20 studies describing LLM-based systems applied to exercise, fitness, sports, and rehabilitation coaching. Each entry includes the model name, application domain, input and output types, datasets used, and evaluation strategies. The table further distinguishes whether the evaluation was supported by user feedback, expert comparisons, or expert scoring. Studies are listed chronologically to reflect the evolution of AI coaching models over time. This table provides a detailed overview of model design and evaluation diversity across various health-related AI coaching scenarios.

**Table 2.** Summary of key features and evaluation of large language model-based models in exercise and health coaching by publication date.

Studies/Year	Model name	Application scenario	Input type	Output type	Datasets	Evaluation metrics	Evaluation outcome
Jörke et al [17], 2025‐03	GPT - Coach	Personalized physical activity plan	Self-tracking data+prompt chaining	Advice, plan, visualizations	16 users, 3-month HealthKit Data	MITI^a^, User Survey, SASSI^b^, versus Vanilla GPT-4	MI^c^-consistent 93%, high personalization (4.6/5)
Li et al [3], 2025‐03	LLaMo	Swing: baseball or golf	3D motion/ video data+prompts	Textual description, QA^d^ answers	6 datasets; 80K motions; 600K QA pairs	MoVid-Bench, BABEL-QA, QA-*F*_1_-score, versus Motion-GPT	*F-*1: 0.458; Swing score: 2.48; MoVid-Bench: Acc^e^ ↑^f^ (55.32%)
Xing et al [4], 2025‐03	LLM - FMS^g^	Functional movement screening	FMS key frames+prompt	Scoring, explanations, suggestions	1812 images (15 movements)	Acc, maF1^h^, Kappa^i^	Acc: 0.91, maF1: 0.87, Kappa: 0.82
Ma et al [18], 2025‐02	N/A	Table tennis coach	Player video+ball trajectory+pose	Error analysis, training advice, strategy	Table tennis dataset	Acc, Expert Rating, Fleiss’ Kappa, versus GPT-4	Acc↑ (67.4%), Fleiss’ Kappa 0.79, Expert: 8.8/10
Yao et al [19], 2025‐03	Count-LLM	Repetitive exercise counting	Video frames+ prompts	Counting number	Rep-Count, UCF-Rep^j^, Countix	MAE^k^, OBO^l^, versus RepNet, TransRAC, ESCounts	RepCount OBO↑0.639; UCFRep OBO↑0.839
Mantena et al [20], 2025‐02	MHC-Coach	Cardiovascular health promotion	TTM^m^ stage+ health data	Motivational coaching messages	3268 expert messages	User preference, expert scores	68% prefer MHC-Coach; Expert Effectiveness 4.4 versus 2.8
Huang et al [21], 2025‐02	N/A	Weight loss	Weight, calorie, activity datas+ prompts	Personalized recommendations	Project ReLearn trial (87 participants)	Helpfulness rating, Misidentification rate	82% AI helpfulness ≥3, 50% misidentified
Saraç et al [22], 2025‐02	N/A	Weight management plans	Detailed prompt	Exercise plans	4 programs (3 AI^n^, 1 expert)	versus ACSM^o^/NASM^p^ guidelines & human experts	AI plans differ from experts; experts are safer
Havers et al [11], 2024‐12	N/A	Hypertrophy plans	Simple/Detailed prompts	Training plans	8 plans / 12 experts	Expert scores, Fleiss’ Kappa	High reprod.; GPT-4>Gemini; Low expert agreement
Ong et al [23], 2024‐09	N/A	Health promotion	Client Qs+ SleepQA retrieval	Advices	SleepQA (1000+ articles)	Acc, Readability, Helpfulness, Empathy, Harm	No sig. diff. (experts, GPT-4); Lay users: LLM^q^ more helpful
Strömel et al [24], 2024‐05	N/A	Fitness tracker data reflection	self-tracking step data (FitBit)+ prompts	Narrative descriptions	273 users’ 7-day step data	TSRI^r^, UES-SF^s^	Focused Attention ↑, Reward ↑, Comparison ↑
Kim et al [25], 2024‐04	Health Alpaca	Wearable-based health prediction	Wearable sensor data+ demographics+prompts	Fitness and health monitoring	PM Data^t^, Life Snaps, GLOBEM^u^, AW FB^v^	Acc., MAE, MAPE^w^, F1 Score	8/10 tasks best performance, outperform GPT-3.5/4
Willms et al [26], 2024‐02	N/A	Physical activity	Behavior data+M-PAC^x^ theory prompts	Personalized physical activity	N/A	Acceptability, ease of use	Acceptable (with expert filtering)
Zaleski et al [27], 2024‐01	N/A	Exercise prescription	Open-ended prompts	Exercise recommendations	ACSM guidelines	Acc, comprehensiveness, readability	Acc.: 90.7%; comprehensiveness: 41.2%
Dergaa et al [28], 2023‐12	N/A	Exercise prescription	Health profiles+prompts	Exercise program	N/A	Expert scoring (FITT^y^ adherence, safety)	Safe but conservative; lacks personalization
Haag et al [29], 2023‐11	N/A	Cardiac rehabilitation	Personal profiles+ prompts	Exercise recommendations	3 Personas ×5 Contexts	Appropriateness, engagement, effectiveness, professionalism	All metrics GPT-4 >HCP^z^ >LayP^aa^; mean Score: 5.47‐5.94/7
Washif et al [12], 2023‐11	N/A	Strength prescription	User-level prompts	Training plans	3 programs	Expert appraisal (qualitative)	GPT-4.0 >GPT-3.5; plans require expert tuning.
Shin et al [30], 2023‐09	N/A	Personalized exercise plan	Goals+availability+obstacles	Personalized plans	75 exercise list+ user study data	User feedback, expert rating	Personalization: 5.83/7; FITT (freq: 5.67, time: 5.06)
Vardhan et al [31], 2023‐04	N/A	Health promotion (Walking)	Self-reports+COM-B^ab^ priming	Coaching responses	PACE^ac^ dataset	User survey (empathy, actionability, etc)	Actionability↑, positive sentiment↑, empathy↑
Sivarajku et al [32], 2023‐03	N/A	Rehabilitation exercise extraction	Clinical notes+few-shot prompts	Exercise concept classification	23,724 rehab notes, 300 annotated	Precision, recall, *F*_1_-score	Few-shot: 0.37; zero-shot: 0.35; best concept: 0.846

a MITI: Motivational Interviewing Treatment Integrity.

b SASSI: Subjective Assessment of System Suitability for Implementation.

c MI: Motivational Interviewing.

d QA: Question Answering.

e Acc: Accuracy rate.

f ↑: indicates superior performance relative to baseline or comparison models.

g FMS: Functional movement screen

h maF1: Macro-averaged F1 score

i Kappa: Cohen or Fleiss agreement score.

j UCF-Ref: University of Central Florida Repetitive Action Dataset

k MAE: mean absolute error.

l OBO: Off-By-One.

m TTM: Transtheoretical Model.

n AI: artificial intelligence.

o ACSM: American College of Sports Medicine.

p NASM: National Academy of Sports Medicine.

q LLM: large language model.

r TSRI: Technology-Supported Reflection Inventory.

s UES-SF: User Engagement Scale-Short Form.

t PMData: Personal Monitoring Data

u GLOBEM: Generalization of Longitudinal BEhavior Modeling

v AW FB: Apple Watch and FitBit

w MAPE: mean absolute percentage error.

x M-PAC: Multi-Process Action Control framework.

y FITT: frequency, intensity, time, type.

z HCP: health care professional.

aa LayP: Layperson.

ab COM-B: Capability, Opportunity, Motivation, Behaviour.

ac PACE: Personalized and Automated Coaching Engine

### Distribution of Models, Applications, and Evaluation Strategies

Table 3 provides an aggregated statistical overview of the foundational models, application domains, and evaluation strategies across the 20 studies. The field is predominantly driven by proprietary models, with 75% (15/20) [11,12,17,18,21-30,32] of studies using OpenAI’s ChatGPT series. In terms of application, research is concentrated in 2 main areas: exercise and health plan generation (8/20, 40%) [11,12,21,22,25,27,28,30] and behavioral and motivational coaching (7/20, 35%) [17,20,23,24,26,29,31]. Evaluation strategies are highly varied: human-rating metrics are the most common approach (16/20, 80%) [3,11,12,17,18,20-24,26-31], typically using expert scoring and user surveys. In contrast, automated performance metrics are used less frequently (8/20, 40%) [3,4,18,19,23,25,31,32], primarily for objective, task-oriented evaluations.

This table categorizes the 20 included studies based on 3 key dimensions. A basic model summarizes the frequency of the underlying LLM architectures used. Focus areas and application domains classify each study into one of 3 primary application clusters based on its core objective. Evaluation strategies report the frequency and proportion of the 3 primary evaluation approaches used across the literature: automated performance metrics, human-rating metrics, and study-design metadata. This comprehensive summary enables readers to quickly understand the dominant model types, primary research applications, and methodological preferences currently adopted in the AI coaching literature.

**Table 3.** Distribution of foundational models, application domains, and evaluation strategies across included studies.

Variable	Values (N=20), n (%)
Basic model	
ChatGPT	15 (75) [11,12,17,18,21-30,32]
Gemini	4 (20) [11,18,22,25]
Llama	3 (15) [20,23,25]
Other models (eg, Vicuna, LaMDA)	2 (10) [19,31]
Unspecified LLM^a^	2 (10) [3,4]
Focus areas and application domains	
Movement analysis and correction	5 (25) [3,4,18,19,32]
Exercise and health plan generation	8 (40) [11,12,21,22,25,27,28,30]
Behavioral and motivational coaching	7 (35) [17,20,23,24,26,29,31]
Evaluation strategies	
Automated performance metrics	8 (40) [3,4,18,19,23,25,31,32]
Human-rating metrics	16 (80) [3,11,12,17,18,20-24,26-31]
Study-design metadata	20 (100) [3,4,11,12,17-27,29-32]

a LLM: large language model.

### Summary

Collectively, these findings paint a picture of a vibrant and rapidly expanding field characterized by significant innovation in application. However, this innovation is juxtaposed with a clear lack of methodological standardization and a general trend toward less rigorous evaluation designs. The heavy reliance on human-rating metrics without consistent reporting of interrater reliability, coupled with a limited use of real-world data and contexts, highlights a critical gap between the technical potential of LLM-based coaches and the current state of their scientific validation.

## Discussion

### Principal Findings

**Figure 2.**
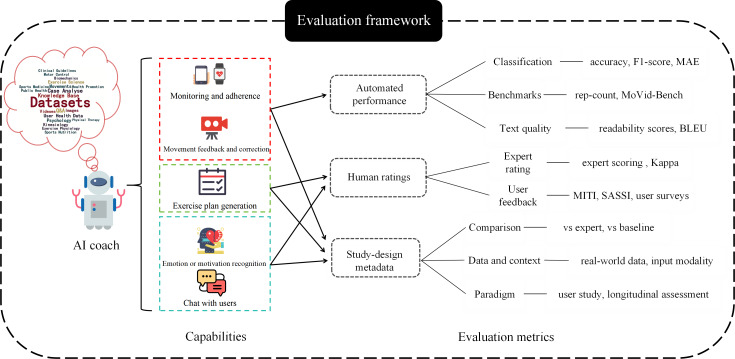
Mapping artificial intelligence coach capabilities to evaluation methodologies. AI: artificial intelligence; BLEU: Bilingual Evaluation Understudy; MAE: mean absolute error; MITI: Motivational Interviewing Treatment Integrity; MoVid-Bench: Motion Video Benchmark; rep-count: Repetition Count; SASSI: Subjective Assessment of System Suitability for Implementation.

A primary contribution of this review is the synthesis of our findings into a conceptual framework designed to guide future evaluation studies (see Figure 2). This framework maps AI coach capabilities to a multidimensional set of evaluation metrics, and our analysis reveals a clear divide in how researchers currently use its different components. The field is largely split between 2 clusters, each focusing on different pillars of the framework. The first, an application-focused cluster, rightly prioritizes the human ratings pillar to assess subjective coaching qualities like personalization and empathy [17,24]. However, as our rigor assessment shows (median ERS of 2.5), this approach is often compromised by a lack of methodological depth, particularly in reporting interrater reliability and using real-world data. The second, a technique-focused cluster, logically centers on the Automated Performance pillar, using objective metrics on benchmark datasets to evaluate discrete tasks such as movement classification [3,4,19]. While strong in their technical validation, these studies often neglect the human-centered and study design components essential for evaluating a holistic coaching experience. This fundamental divide highlights a critical gap: the scarcity of studies that adopt a truly multidimensional approach as advocated by our framework, which would integrate the objective rigor of technical benchmarks with the ecological validity of human-centered validation.

This figure illustrates how an AI coach, powered by a diverse set of datasets and a knowledge base (far left), performs key capabilities (center left) like movement correction and plan generation. These capabilities are then mapped to 3 distinct categories of evaluation methods (right): automated performance metrics, human ratings, and study design metadata. The framework emphasizes a holistic assessment strategy, combining objective metrics with human-centered feedback and rigorous study paradigms.

### Strengths and Weaknesses Within the Evaluation Landscape

The strengths of the current evaluation landscape lie within these distinct, albeit siloed, approaches. Expert-driven assessments, for example, are invaluable for ensuring the scientific validity and clinical relevance of AI-generated content, particularly in aligning exercise plans with established guidelines [22,27,28]. Similarly, user-centered assessments using validated tools like the MITI scale provide critical insights into usability and engagement that automated metrics cannot capture [17,21]. On the other hand, technical evaluations using benchmarks provide objective and reproducible quantification for specific tasks like movement analysis, which is a strength that subjective ratings lack [3,19].

However, as our rigor assessment highlights, these strengths are often undermined by significant and widespread limitations. The subjectivity inherent in human-centered evaluations becomes a critical weakness when not properly validated; fewer than half of the studies (n=9, 45%) reported interrater reliability, casting doubt on the consistency of their findings. Furthermore, the lack of ecological validity is a major concern, with only 40% of studies using real-world contexts or user-generated data. This reliance on hypothetical scenarios severely constrains the generalizability of findings. Finally, while objective automated metrics are a strength in terms of reproducibility, their narrow focus is a limitation, as they often fail to capture the broader behavioral and psychological dimensions of coaching effectiveness, such as motivation or long-term adherence [3,25]. In contrast, the few studies that achieved a high ERS successfully integrated these multiple dimensions, demonstrating a potential pathway for establishing the robust and clinically meaningful benchmarks that the field currently lacks [17,24].

### Mitigating Risks in AI Coaching

While our review highlights the potential of LLM-based coaches, their practical and ethical deployment in digital health is contingent upon addressing critical challenges of data privacy, explainability, and bias, which issues were only touched upon by the included studies. The use of personal health data, especially multimodal inputs such as sensor readings and video, raises significant privacy concerns. This is particularly acute when using proprietary models that process data on third-party servers, creating challenges for compliance with stringent data protection regulations like General Data Protection Regulation [33,34] or ISO/ie,C 27701 (privacy and security in the era of AI) [35].

Furthermore, the inherent “black-box” nature of many LLMs presents a major barrier to explainability, eroding the trust of both users and clinicians who need to understand the rationale behind an exercise prescription [5,36]. Finally, these models can inadvertently perpetuate or even amplify societal biases present in their training data, potentially leading to inequitable or unsafe recommendations for certain demographics, body types, or individuals with disabilities [37].

Addressing these challenges is essential for the responsible development of the field. To mitigate privacy risks and ensure regulatory compliance, future research should prioritize the evaluation of systems built on open-source models that can be deployed locally or in secure, Health Insurance Portability and Accountability Act-compliant environments. Techniques like federated learning represent a critical pathway for training models on decentralized user data without compromising privacy [38]. To enhance explainability and trust, the integration of Retrieval-Augmented Generation (RAG) is a promising strategy. RAG can ground AI-generated advice in verifiable, evidence-based sources, such as American College of Sports Medicine (ACSM) guidelines or peer-reviewed literature, making the model’s reasoning more transparent and reducing the risk of factual “hallucinations” [39,40]. Finally, to combat bias, a human-centered approach is imperative. This includes implementing systematic bias auditing and “red teaming” as a standard part of the evaluation process and, crucially, adopting co-design methodologies that involve diverse and representative user groups in the design and testing phases to ensure AI coaching systems are equitable, inclusive, and truly personalized [41,42].

### Future Directions

Based on our findings and promising advancements in evaluation science, we propose a 2-pronged approach for future research focused on enhancing the reliability and efficiency of AI coach assessments.

First, the most immediate priority is to address the foundational challenge of factual accuracy and trustworthiness in AI-generated advice. The integration of RAG presents a critical pathway to mitigate the risks of hallucination and ensure that coaching recommendations are grounded in evidence. Future work should move beyond simply applying RAG and focus on a structured implementation and validation process [39]. For developers, this involves the crucial task of curating and maintaining high-quality, domain-specific knowledge bases from gold-standard sources like ACSM guidelines and peer-reviewed literature. For researchers, the focus must be on establishing new evaluation metrics tailored for RAG systems. This includes not only assessing the final output but also the factual consistency between the generated text and the retrieved source documents [40]. Furthermore, applying structured evaluation frameworks such as SCORE (Safety, Clinical Consensus, Objectivity, Reproducibility, Explainability) could significantly enhance the reliability and transparency of AI-generated coaching recommendations.

Second, to overcome the scalability bottlenecks of current human-rating methods, future research should explore novel evaluation frameworks like the Adaptive Precise Boolean Framework. This approach addresses the limitations of Likert scales (eg, low interrater reliability and time-consuming) by deconstructing complex evaluation criteria into a series of simple, granular, and objective Yes/No questions [43]. The implementation of this approach could follow a 2-stage process. Initially, for researchers and clinicians, the task is to collaborate in developing standardized, expert-validated “Precision Boolean rubrics” for core coaching domains (eg, exercise plan safety and motivational feedback quality). Once these robust rubrics are established, the next step is to explore the use of LLMs as automated evaluators to perform the binary (Yes/No) judgment. As recent work suggests, this method not only yields substantially higher interrater agreement among both expert and nonexpert evaluators but also has the potential to halve the evaluation time while maintaining high-quality assessment [43]. Adopting such a framework would provide a scalable, efficient, and reliable mechanism for assessing the safety and quality of AI coaching at scale, paving the way for more rigorous and comparable studies in the future.

While the proposed multidimensional framework represents an ideal standard, we acknowledge that its full implementation poses significant practical challenges. These include substantial resource requirements for conducting rigorous user studies, the need for interdisciplinary expertise, and barriers to clinical adoption and data collection. To bridge this gap between the ideal and the practical, we advocate for a phased approach. Initial feasibility studies could prioritize automated benchmarks and expert-driven assessments, while later-stage, well-funded research should aim for longitudinal studies incorporating validated user-centered metrics in real-world settings. Furthermore, developing shared resources, such as standardized datasets and evaluation protocols, could lower the barrier to entry for researchers and foster more robust and comparable evidence across the field.

### Limitations of the Study

This review has several limitations. First, a critical limitation is the exclusion of commercial AI coaching systems, like WHOOP Coach (WHOOP, Inc), ONVY (ONVY HealthTech Group GmbH), and S.A.R.A.H (World Health Organization), due to the lack of publicly available technical documentation. This may significantly skew our findings, as these systems dominate real-world applications and may use more advanced evaluation methods not visible in academic literature. Second, our inclusion criteria, which required studies to have clearly reported evaluation strategies, may have led to the exclusion of promising models that lacked formal evaluation documentation at the time of our search. Third, the ERS used in this review is a custom tool developed specifically for this study’s context and has not been externally validated. While it provides a structured framework for appraisal, the scores should be interpreted as indicators of methodological depth rather than absolute measures of quality. Fourth, the heterogeneity in datasets, evaluation metrics, and methods across the included studies limits the direct comparability and generalizability of the findings. Fifth, many multimodal models were assessed using static or synthetic data, which may not accurately reflect their real-time performance in complex, real-world environments. Finally, none of the studies evaluated long-term outcomes, such as sustained behavior change or physical improvements, which are critical for assessing the true impact and effectiveness of AI coaches.

### Conclusions

Evaluating LLM-based exercise and health coaches demands a multifaceted strategy, yet our review reveals a fragmented landscape characterized by a lack of standardization and methodological rigor. This fragmentation is largely driven by a dichotomy between application-focused studies, which prioritize subjective human ratings, and technique-focused studies, which rely on objective automated metrics. To bridge this divide, this review synthesizes these disparate approaches into a unified conceptual framework that advocates for integrating the objective rigor of technical benchmarks with the ecological validity of human-centered validation. Future progress hinges on implementing this integrated approach. Two critical pathways are (1) the integration of RAG to ensure factual accuracy, and (2) the adoption of scalable and reliable evaluation paradigms, such as the Adaptive Precise Boolean Framework, to overcome the limitations of current methods. However, it is important to interpret these findings with caution, as this review is limited by the heterogeneity of the included studies and the early-stage nature of the field, where most evaluations have yet to assess long-term behavioral outcomes in real-world settings.

## Supplementary material

10.2196/79217 Multimedia Appendix 1 Detailed search strategies for the scoping review, including search strings, filters, and results for all searched databases. The search was conducted on July 31, 2025, for the period between March 1, 2023, and July 31, 2025.

10.2196/79217 Checklist 1 PRISMA 2020 abstract checklist.

10.2196/79217 Checklist 2 PRISMA 2020 checklist.

## Abbreviations

ACSM: American College of Sports Medicine
AI: artificial intelligence
CC-BY 4.0 license: Creative Commons Attribution 4.0 International License
ERS: Evaluation Rigor Score
LLM: large language model
MITI: Motivational Interviewing Treatment Integrity
PRISMA: Preferred Reporting Items for Systematic Reviews and Meta-Analyses
PRISMA-ScR: Preferred Reporting Items for Systematic Reviews and Meta-Analyses extension for Scoping Reviews
RAG: Retrieval-Augmented Generation
SASSI: Subjective Assessment of System Suitability for Implementation
SCORE: Safety, Clinical Consensus, Objectivity, Reproducibility, Explainability

## References

[1] Noh E, Won J, Jo S, Hahm DH, Lee H (2023). Conversational agents for body weight management: systematic review. J Med Internet Res.

[2] Soenksen LR, Ma Y, Zeng C (2022). Integrated multimodal artificial intelligence framework for healthcare applications. NPJ Digit Med.

[3] Li L, Jia S, Wang J, Dai J Human motion instruction tuning.

[4] Xing Q, Xing X, Guo P, Tang Z, Shen Y (2025). LLM-FMS: a fine-grained dataset for functional movement screen action quality assessment. PLoS ONE.

[5] Chang Y, Wang X, Wang J (2024). A survey on evaluation of large language models. ACM Trans Intell Syst Technol.

[6] Xia H, Yang Z, Wang Y SportQA: a benchmark for sports understanding in large language models.

[7] Ge K, Chen L, Zhang K, Luo Y, Shi T, Fan L (2024). SCBench: a sports commentary benchmark for video llms. arXiv.

[8] Xia H, Yang Z, Zou J, Tracy R, Wang Y, Lu C (2024). Sportu: a comprehensive sports understanding benchmark for multimodal large language models. arXiv.

[9] Den Hartigh RJR, Meerhoff LRA, Van Yperen NW (2024). Resilience in sports: a multidisciplinary, dynamic, and personalized perspective. Int Rev Sport Exerc Psychol.

[10] Li L, Chen G, Shi H, Xiao J, Chen L (2024). A survey on multimodal benchmarks: in the era of large AI models. arXiv.

[11] Havers T, Masur L, Isenmann E (2025). Reproducibility and quality of hypertrophy-related training plans generated by GPT-4 and Google Gemini as evaluated by coaching experts. Biol Sport.

[12] Washif JA, Pagaduan J, James C, Dergaa I, Beaven CM (2024). Artificial intelligence in sport: exploring the potential of using ChatGPT in resistance training prescription. Biol Sport.

[13] Kamel S, Caputo J, Johnson S (2025). ChatGPT-generated resistance training programs. ACSM’s Health and Fitness Journal.

[14] Arksey H, O’Malley L (2005). Scoping studies: towards a methodological framework. Int J Soc Res Methodol.

[15] Tricco AC, Lillie E, Zarin W (2018). PRISMA Extension for Scoping Reviews (PRISMA-ScR): checklist and explanation. Ann Intern Med.

[16] Pham MT, Rajić A, Greig JD, Sargeant JM, Papadopoulos A, McEwen SA (2014). A scoping review of scoping reviews: advancing the approach and enhancing the consistency. Res Synth Methods.

[17] Jörke M, Sapkota S, Warkenthien L (2025). GPTCoach: towards LLM-based physical activity coaching.

[18] Ma W, Liu Y, Yi Q (2025). Table tennis coaching system based on a multimodal large language model with a table tennis knowledge base. PLoS One.

[19] Yao Z, Cheng X, Huang Z, Li L, Yao Z, Cheng X, Huang Z, Li L (2025). CountLLM: towards generalizable repetitive action counting via large language model.

[20] Mantena S, Johnson A, Oppezzo M (2025). Fine-tuning large language models in behavioral psychology for scalable physical activity coaching. medRxiv.

[21] Huang Z, Berry MP, Chwyl C, Hsieh G, Wei J, Forman EM (2025). Comparing large language model AI and human-generated coaching messages for behavioral weight loss. J technol behav sci.

[22] Saraç H, Ulusoy İT, Alpay J, Ödemiş H, Söğüt M (2025). Evaluating the potential role of AI chatbots in designing personalized exercise programs for weight management. Int J Hum Comput Interact.

[23] Ong QC, Ang CS, Chee DZY (2024). Advancing health coaching: a comparative study of large language model and health coaches. Artif Intell Med.

[24] Strömel KR, Henry S, Johansson T, Niess J, Woźniak PW, Stroemel KR, Henry S, Johansson T, Niess J, Wozniak PW Narrating fitness: leveraging large language models for reflective fitness tracker data interpretation.

[25] Kim Y, Xu X, McDuff D, Breazeal C, Park HW (2024). Health-LLM: large language models for health prediction via wearable sensor data. arXiv.

[26] Willms A, Liu S (2024). Exploring the feasibility of using ChatGPT to create just-in-time adaptive physical activity mHealth intervention content: case study. JMIR Med Educ.

[27] Zaleski AL, Berkowsky R, Craig KJT, Pescatello LS (2024). Comprehensiveness, accuracy, and readability of exercise recommendations provided by an AI-based chatbot: mixed methods study. JMIR Med Educ.

[28] Dergaa I, Saad HB, El Omri A (2024). Using artificial intelligence for exercise prescription in personalised health promotion: a critical evaluation of OpenAI’s GPT-4 model. Biol Sport.

[29] Haag D, Kumar D, Gruber S The last JITAI? exploring large language models for issuing just-in-time adaptive interventions: fostering physical activity in a prospective cardiac rehabilitation setting.

[30] Shin D, Hsieh G, Kim YH (2023). PlanFitting: tailoring personalized exercise plans with large language models. arXiv.

[31] Hegde N, Vardhan M, Nathani D (2024). Infusing behavior science into large language models for activity coaching. PLOS Digit Health.

[32] Sivarajkumar S, Gao F, Denny P (2024). Mining clinical notes for physical rehabilitation exercise information: natural language processing algorithm development and validation study. JMIR Med Inform.

[33] Yachou Y (2022). AI Solutions in Healthcare: GDPR Compliance and the New AIA.

[34] Ogobegwu JN (2024). General Data Protection Regulation (GDPR) and Its Impact on the Development of Artificial Intelligence (AI) in Marketing.

[35] Ranjbar A, Mork EW, Ravn J (2024). Managing risk and quality of AI in healthcare: are hospitals ready for implementation?. Risk Manag Healthc Policy.

[36] Bharati S, Mondal MRH, Podder P (2023). A review on explainable artificial intelligence for healthcare: why, how, and when?. IEEE Trans Artif Intell.

[37] Weidinger L, Mellor J, Rauh M, Griffin C, Uesato J, Huang PS (2021). Ethical and social risks of harm from language models. arXiv.

[38] Xu J, Glicksberg BS, Su C, Walker P, Bian J, Wang F (2021). Federated learning for healthcare informatics. J Healthc Inform Res.

[39] Ke YH, Jin L, Elangovan K (2025). Retrieval augmented generation for 10 large language models and its generalizability in assessing medical fitness. NPJ Digit Med.

[40] Wu J, Zhu J, Qi Y, Menolascina F Medical graph RAG: evidence-based medical large language model via graph retrieval-augmented generation.

[41] Chang CT, Farah H, Gui H (2025). Red teaming ChatGPT in medicine to yield real-world insights on model behavior. NPJ Digit Med.

[42] Panigutti C, Beretta A, Fadda D (2023). Co-design of human-centered, explainable AI for clinical decision support. ACM Trans Interact Intell Syst.

[43] Mallinar N, Heydari AA, Liu X, Faranesh AZ, Winslow B, Hammerquist N (2025). A scalable framework for evaluating health language models. arXiv.

