# Supplementary material for: Evaluation Strategies for Large Language Model-Based Models in Exercise and Health Coaching: Scoping Review
Source: J Med Internet Res. 2025 Oct 14;27:e79217. doi: 10.2196/79217 (PMC12520646; doi:10.2196/79217)
Supplement: Multimedia Appendix 1 [file jmir-v27-e79217-s001.docx]

**Multimedia Appendix 1.** Detailed Search Strategies.

1. **Basic Information**

| **Item** | **Content** |
| --- | --- |
| Review Title | Evaluation Strategies for LLM-Based Models in Exercise and Health Coaching: A Scoping Review |
| Author/Searcher | Xiangxun Lai |
| Search Date | 2025-07-31 |
| Databases | PubMed, Web of Science, Google Scholar, arXiv, medRxiv, and bioRxiv |
| Time Range | 2023-03-01 to 2025-07-31 |
| Language Restriction | English only |

1. **Search Records Form**

| **Database** | **Search String** | **Filters Applied** | **Results** |
| --- | --- | --- | --- |
| PubMed | (("large language model"[Title/Abstract] OR "LLM"[Title/Abstract] OR "AI coach"[Title/Abstract] OR "generative AI"[Title/Abstract])) AND (("exercise"[Title/Abstract] OR "fitness"[Title/Abstract] OR "coaching"[Title/Abstract] OR "rehabilitation"[Title/Abstract] OR "movement"[Title/Abstract] OR "physical activity"[Title/Abstract] OR "training"[Title/Abstract])) AND (("evaluation"[Title/Abstract] OR "validation"[Title/Abstract] OR "benchmark"[Title/Abstract] OR "accuracy"[Title/Abstract] OR "performance"[Title/Abstract] OR "metric"[Title/Abstract])) | Publication Dates: 2023/03/01 to 2025/07/31; Language: English. | 745 |
| Google Scholar | allintitle: ("large language model" OR LLM OR "AI coach" OR "generative AI") (exercise OR fitness OR coaching OR rehabilitation OR movement OR "physical activity" OR training) | Timeframe: 2023/03/01 to 2025/07/31. Non-English results were excluded manually during screening. | 408 |
| Web of Science | TS=(large language model OR LLM) AND TS=(exercise OR fitness OR coaching OR rehabilitation OR movement OR human pose OR physical activity OR fitness training) AND TS=(evaluation OR validation OR benchmark OR accuracy OR performance OR metric) | Publication Dates: March 2023 to March 2025; Language: English. | 1252 |
| arXiv | Query: [order: -announced_date_first; size: 50; date_range: from 2023-03-01 ; include_cross_list: True; terms: AND title=(large language model OR LLM) AND (exercise OR coaching OR fitness OR rehabilitation OR human pose OR movement); AND abstract=(large language model OR LLM) AND (exercise OR coaching OR fitness OR rehabilitation OR human pose OR movement)](https://arxiv.org/search/advanced?terms-0-operator=AND&terms-0-term=(large+language+model+OR+LLM)+AND+(exercise+OR+coaching+OR+fitness+OR+rehabilitation+OR+human+pose+OR+movement)&terms-0-field=title&terms-1-operator=AND&terms-1-term=(large+language+model+OR+LLM)+AND+(exercise+OR+coaching+OR+fitness+OR+rehabilitation+OR+human+pose+OR+movement)&terms-1-field=abstract&classification-physics_archives=all&classification-include_cross_list=include&date-year=&date-filter_by=date_range&date-from_date=2023-03-01&date-to_date=&date-date_type=submitted_date&abstracts=show&size=50&order=-announced_date_first) | Submission date: 2023/03/01 to 2025/07/30. | 428 |
| medRxiv and bioRxiv | for term "("large language model" OR LLM) AND (exercise OR coaching OR fitness OR rehabilitation OR movement OR wellness)" and abstract or title "("large language model" OR LLM) AND (exercise OR coaching OR fitness OR rehabilitation OR movement OR wellness)" (match all words) | Submission date: 2023/03/01 to 2025/07/30. | 314 |

Caption:

Detailed search strategies for the scoping review, including search strings, filters, and results for all searched databases. The search was conducted on July 31, 2025, for the period between March 1, 2023, and July 31, 2025.
